# Psycholinguistic norms for more than 300 lexical signs in German Sign Language (DGS)

**DOI:** 10.3758/s13428-020-01524-y

**Published:** 2021-02-11

**Authors:** Patrick C. Trettenbrein, Nina-Kristin Pendzich, Jens-Michael Cramer, Markus Steinbach, Emiliano Zaccarella

**Affiliations:** 1 Department of Neuropsychology, Max Planck Institute for Human Cognitive & Brain Sciences, Stephanstraße 1a, Leipzig, 04103 Germany; 2 International Max Planck Research School on Neuroscience of Communication: Structure, Function, & Plasticity (IMPRS NeuroCom), Stephanstraße 1a, Leipzig, 04103 Germany; 3 SignLab, Department of German Philology, Georg-August-University, Käte-Hamburger-Weg 3, Göttingen, 37073 Germany

**Keywords:** German Sign Language, Visuo-spatial modality, Subjective ratings, Lexical frequency, Age of acquisition, Iconicity, Transparency

## Abstract

Sign language offers a unique perspective on the human faculty of language by illustrating that linguistic abilities are not bound to speech and writing. In studies of spoken and written language processing, lexical variables such as, for example, age of acquisition have been found to play an important role, but such information is not as yet available for German Sign Language (*Deutsche Gebärdensprache*, DGS). Here, we present a set of norms for frequency, age of acquisition, and iconicity for more than 300 lexical DGS signs, derived from subjective ratings by 32 deaf signers. We also provide additional norms for iconicity and transparency for the same set of signs derived from ratings by 30 hearing non-signers. In addition to empirical norming data, the dataset includes machine-readable information about a sign’s correspondence in German and English, as well as annotations of lexico-semantic and phonological properties: one-handed vs. two-handed, place of articulation, most likely lexical class, animacy, verb type, (potential) homonymy, and potential dialectal variation. Finally, we include information about sign onset and offset for all stimulus clips from automated motion-tracking data. All norms, stimulus clips, data, as well as code used for analysis are made available through the Open Science Framework in the hope that they may prove to be useful to other researchers: 10.17605/OSF.IO/MZ8J4

## Introduction

Sign languages provide the unique opportunity to ask questions about the human language faculty that go beyond considerations bound to language in its spoken and written form. In the past decades, increased interest in sign language in theoretical linguistics has revealed deep similarities between spoken and sign language despite the striking differences between the auditory-oral and visuo-spatial modality (Aronoff, Meir, & Sandler, 2005; Baker, van den Bogaerde, Pfau, & Schermer, 2016; Cecchetto, 2017; Mathur & Rathmann, 2014; Meier, 2012; Pfau, Salzmann, & Steinbach, 2018; Sandler & Lillo-Martin, 2001, 2008; Wilbur, 2012). This theoretical interest has been accompanied by an upsurge of psycholinguistic and neurolinguistic studies on sign language processing in different labs around the world, thus contributing to a deeper level of understanding of the human capacity for language. These studies have revealed important similarities and differences between modalities with regard to both psychological processes (Gutiérrez, Müller, Baus, & Carreiras, 2012; Gutiérrez, Williams, Grosvald, & Corina, 2012; Hosemann, Herrmann, Sennhenn-Reulen, Schlesewsky, & Steinbach, 2018; Vinson, Thompson, Skinner, Fox, & Vigliocco, 2010) and the underlying neural representation (Emmorey, 2015; MacSweeney, Capek, Campbell, & Woll, 2008; Trettenbrein, Papitto, Friederici, & Zaccarella, 2021).

Reaction time studies, eye tracking experiments, and electroencephalography investigations of spoken and written and, more recently, sign language processing have underlined the importance of controlling for lexical variables such as frequency and age of acquisition (AoA) in psycholinguistic experiments. Just like speakers, signers are known to show sensitivity to these lexical variables during sign language processing (e.g., Carreiras, Gutiérrez-Sigut, Baquero, & Corina, 2008; Emmorey, Petrich, & Gollan, 2013; Gutiérrez, Williams, et al., 2012). The public availability of many large-scale corpora containing item-specific lexical variables in different spoken and written languages has drastically increased (e.g., CELEX for English, Dutch, and German, Baayen, Piepenbrock, & Gulikers, 1995; LEXIQUE for French, New, Pallier, Brysbaert, & Ferrand, 2004; dlexDB for German, Heister et al., 2011, SUBTLEX for Dutch, American English, Chinese, Spanish, German, Greek, British English, Polish, and Italian; available from http://crr.ugent.be). The creation of corpora of a similar scale for sign language is inherently more difficult due to modality-dependent differences in the way video data are collected and can be (semi-)automatically processed (Quer & Steinbach, 2019). Nevertheless, a number of ongoing projects at varying stages of public availability are now emerging, for example, the New Zealand Sign Language (NZSL) corpus project (McKee & Kennedy, 2006), the British Sign Language (BSL) corpus project (Schembri, Fenlon, Rentelis, & Cormier, 2017), and, more recently, the German Sign Language (*Deutsche Gebärdensprache*, DGS) corpus project (“DGS-Korpus”, see https://www.sign-lang.uni-hamburg.de/dgs-korpus). However, compared to large-scale corpora of spoken and written languages, the emerging sign language corpora are still limited with regard to their psycholinguistic applicability.

An alternative effective approach to the creation of psycholinguistic norms for sign language is that of using subjective ratings by deaf signers. Normed sign language datasets derived from subjective ratings for different psycholinguistic variables have been made available for BSL (Vinson, Cormier, Denmark, Schembri, & Vigliocco, 2008), American Sign Language (ASL; Caselli, Sehyr, Cohen-Goldberg, & Emmorey, 2017; Mayberry, Hall, & Zvaigzne, 2014), and Spanish Sign Language *(Lengua de Señas Espanõla*, LSE; Gutiérrez-Sigut, Costello, Baus, & Carreiras, 2016). Such subjective ratings have been shown to be reproducible (Gilhooly & Gilhooly, 1980) and are correlated with measures derived from corpus data for both, spoken and signed languages (Balota, Pilotti, & Cortese, 2001; Fenlon, Schembri, Rentelis, Vinson, & Cormier, 2014). The subjective-rating approach has also been successfully employed to create norms for spoken and written languages, for example, providing information about lexical variables for English (Cortese & Fugett, 2004; Cortese & Khanna, 2008; Gilhooly & Logie, 1980; Stadthagen-Gonzalez & Davis, 2006) and German (Schröder, Gemballa, Ruppin, & Wartenburger, 2012).

Here we used the subjective-rating approach to create the first-to-date psycholinguistic norms for more than 300 lexical signs in DGS. These include norms for (1) iconicity, (2) AoA, and (3) frequency derived from ratings by 32 deaf native DGS signers. We also include norms for (4) transparency and (5) iconicity derived from ratings by 30 hearing non-signers who had no knowledge of and prior experience with DGS or any other sign language. The norms for frequency and AoA in the present work are supposed to complement the current lack of corpus-based measures for these lexical variables and will make it possible to control for these variables in psycholinguistic research using DGS stimuli. Concurrently, our norms also provide ratings for iconicity by deaf signers and hearing non-signers as well as an empirical measure of a sign’s transparency to non-signers. In addition, we include a set of annotations for every sign (e.g., one vs. two-handed, place of articulation, most likely lexical category, etc.) in machine-readable form, as well as data from automated motion-tracking of the provided stimulus clips.

**Fig. 1:**
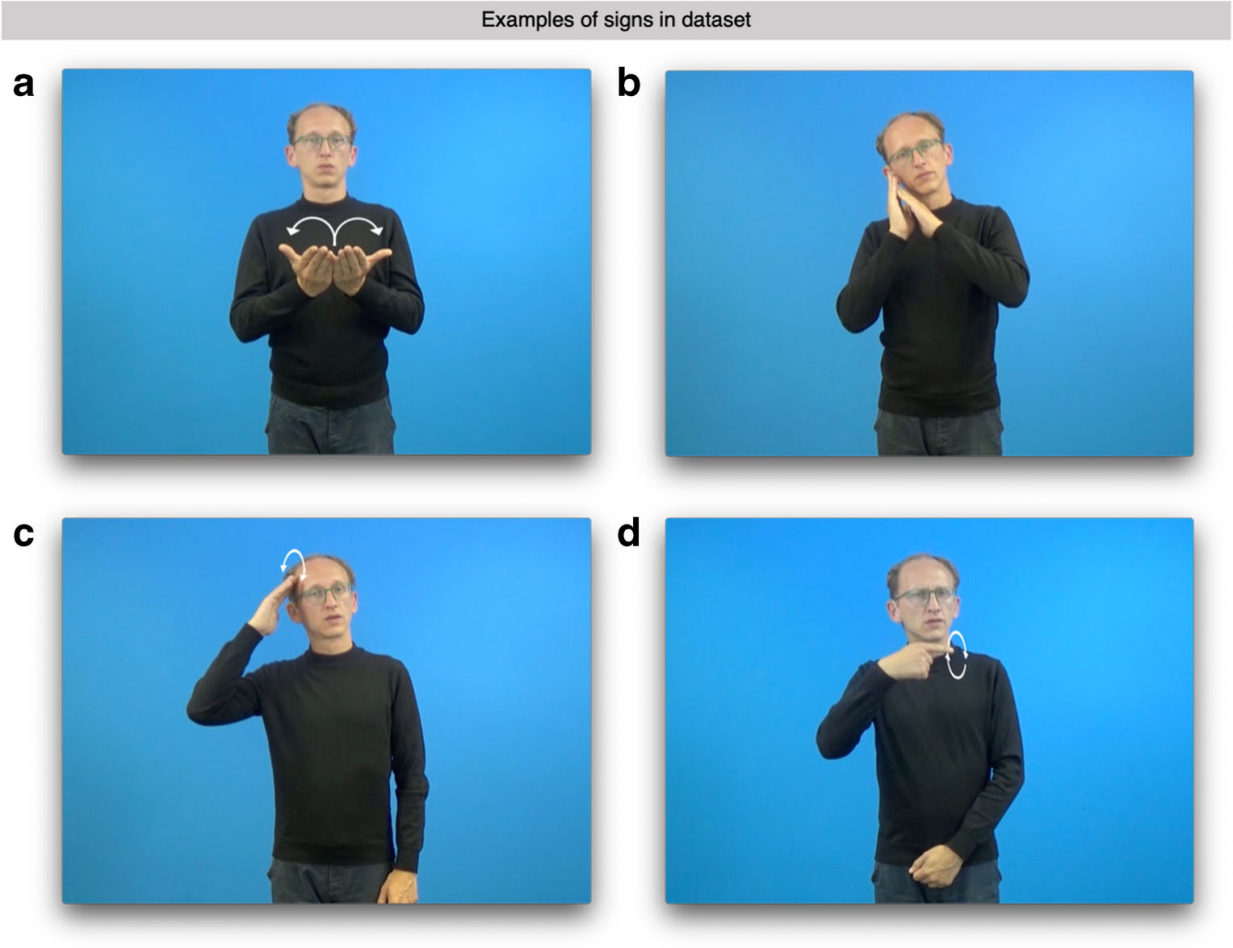
Representative still images of DGS signs with high and low iconicity and transparency that were recorded as part of the normed stimulus set. *White arrows* indicate the sign’s defining path movement. **a** Iconic and only semi-transparent sign BOOK. **b** The iconic and transparent sign SLEEP. **c** Non-iconic and non-transparent sign BOY. **d** The non-iconic and non-transparent sign LIE

As for iconicity, the fact that many signs across different sign languages exhibit iconic properties has already been recognized in early research on sign language (e.g., Klima et al., 1979). By definition, a sign is considered iconic if its form resembles or depicts the form of the referent to a certain extent (Klann, 2014; Liddell, 2003; Schlenker, 2018; Taub, 2001, 2012), as is the case for the sign BOOK depicted in Fig. 1a. It has been estimated that about one third of all lexical signs are iconic (Boyes-Braem, 1986), as the visuo-spatial modality of sign language seems to allow for iconicity to a larger extent than the modalities of spoken and written language. In speech and writing, iconicity is widely considered a marginal phenomenon that is only present in onomatopoeia and sound symbolism (but see Blasi, Wichmann, Hammarström, Stadler, & Christiansen, 2016; Dingemanse, 2013; and Fischer, 2014 for diverging views on the prevalence of iconicity in spoken and written language). Although iconicity is an inherent feature of sign languages, neuroimaging studies have shown that this potential modality-dependent difference does not lead to a distinct neural representation of iconic signs (Emmorey et al., 2004; Klann, Kastrau, & Huber, 2005). From a psycholinguistic point of view, iconicity is now considered a means for establishing a structured mapping between two mental representations with varying degrees of overlap (Emmorey, 2014). Some behavioral studies of iconicity in sign language processing have shown an influence of iconicity in semantic tasks (e.g., Thompson, Vinson, & Vigliocco, 2009) which, however, was absent in studies that did not specifically tap into such structured mappings (Bosworth & Emmorey, 2010; Emmorey, 2014). Regarding sign language acquisition, the role of iconicity has been subject to debate with most researchers now agreeing that the effect of iconicity is at best subtle, as the ability to recognize a sign’s iconic motivation depends on the development of non-linguistic capacities (Emmorey, 2014; Klima et al., 1979; Meier, 2016; Ortega, 2017). Still, iconicity may have an initially faciliatory effect on the conceptual-semantic aspect of sign language acquisition when a sign language is acquired as a second language only later in life (Ortega, 2017; Ortega, Özyürek, & Peeters, 2019).

Crucially, measures for iconicity cannot be derived from corpus data, in contrast to information about lexical frequency and AoA. Early work on sign language by Klima et al. (1979) already proposed that there may be different levels of iconicity, depending on the degree to which a sign’s form and meaning is accessible to signers and non-signers (see also Emmorey, 2014). For example, signers judge equally iconic signs in a foreign sign language as less iconic than the corresponding sign in their native sign language, indicating that lexical accessibility of a sign has an impact on its degree of perceived iconicity (Omardeen, 2018, 2019). Similarly, iconicity may make signs more or less transparent to non-signers: On one end of the spectrum there are rather transparent signs (e.g., BOOK, Fig. 1a; SLEEP Fig. 1b), whereas the other end is occupied by opaque signs (e.g., BOY, Fig. 1c; LIE, Fig. 1d). To make our norms maximally useful for research on DGS with participants who are signers as well as participants who do not know DGS we therefore collected subjective ratings of iconicity from deaf signers as well as hearing non-signers. Collecting data from signers and non-signers allowed us to determine the influence that sign language acquisition has on the awareness and recognition of iconicity in a visuo-spatial language. Lastly, we also include the transparency of a sign as an additional measure of the accessibility of a sign’s meaning to non-signers. These transparency scores were derived from guesses of a sign’s meaning by hearing non-signers (see Pendzich, 2020 for a similar task regarding nonmanual markings). In the context of the present study, a sign’s transparency thus simply reflects the number of correct guesses which, following Klima et al. (1979), we take to be directly related to a sign’s degree of iconicity.

AoA (i.e., the age at with a speaker or signer first acquired a particular word or sign) is another lexical variable that has been studied in many psycholinguistic studies of spoken and written language (e.g., Brysbaert & Ghyselinck, 2006; Cortese & Khanna, 2007; Gilhooly & Gilhooly, 1980; Morrison, Chappell, & Ellis, 1997). The exact role that AoA plays in language processing has been subject to extensive debate, with most researchers now agreeing that AoA is a good predictor of language processing effects if other variables are controlled for (Brysbaert & Ghyselinck, 2006; Cortese & Khanna, 2007). In principle, measures for AoA for any word or sign in any language could also be derived from corpus data of child language acquisition. However, because such data on acquisition is not available for many languages, spoken or signed, researchers studying language processing have frequently employed a subjective-rating approach for creating norms for AoA (Cortese & Khanna, 2008; Gilhooly & Logie, 1980; Schröder et al., 2012; Stadthagen-Gonzalez & Davis, 2006; Vinson et al., 2008). Similar to observations about subjective ratings for lexical frequency, ratings for AoA collected from adults have been shown to be valid estimates of the actual age at which a word is acquired (Gilhooly & Gilhooly, 1980; Morrison et al., 1997). Consequently, we here adopted the strategy pursued by Vinson et al. (2008) for BSL and collected subjective ratings of AoA as a proxy to actual measures of AoA derived from studies of child language by asking subjects to indicate the age range at which they have acquired a particular sign. Lastly, when discussing the role of AoA in sign language research it is of special importance to note that, in many cases, sign language acquisition may be delayed as deaf children may not have access to sign language early in infancy because they were not born to parents who are deaf signers and were not offered sign language input from birth onwards (Quer & Steinbach, 2019). Such children may only have been exposed to DGS in a day nursery, kindergarten, and/or school setting (Meier, 2016). A distribution of subjective ratings of AoA for sign languages may therefore be expected to be slightly right-skewed in comparison to ratings of AoA for spoken languages.

With respect to lexical frequency, subjects’ sensitivity to the relative occurrence of lexical items in linguistic corpora has been heavily studied in spoken and written language processing (see Brysbaert & New, 2009 for a discussion of these measures and the effect of corpus size). Signers have been shown to be sensitive to the lexical frequency of signs, for example, lexical decision and naming times are shorter for high- than low-frequency signs (Carreiras et al., 2008; Emmorey et al., 2013). One strategy for approximating corpus measures of lexical frequency has been to collect subjective ratings of the familiarity of a sign as a proxy to its actual lexical frequency (Vinson et al., 2008). While the relationship between familiarity and frequency remains subject to debate, the difference between familiarity and frequency seems to be small (Stadthagen-Gonzalez & Davis, 2006) so that subjective familiarity approximates measures of frequency well. This is reflected by the fact that subjective ratings of familiarity for BSL are highly correlated with lexical frequency in the BSL corpus (Fenlon et al., 2014). Accordingly, we here adopted the strategy pursued by Caselli et al. (2017) and consider familiarity and frequency as practically indistinguishable. Hence, we collected subjective frequency ratings by directly asking participants to indicate the frequency with which they see a sign being used.

Lastly, the present norms also include a set of machine-readable annotations for every sign which we hope will make the handling of the present data and stimulus video clips more convenient for researchers. For example, we include information about a sign’s most likely German and English correspondences, potential homonyms, the most likely lexical category, and the place of articulation. Given that variation is also an important factor in sign language research (Schembri & Johnston, 2012; for DGS see Langer, 2012, 2018), we also coded salient dialectal variation on the lexical level in the dataset. Moreover, for signs that can receive a verbal interpretation, we indicated whether the verb shows spatial agreement or not. Signs in our dataset which can receive a nominal interpretation have been coded for animacy. This linguistic information is supplemented by information derived from automated motion tracking of the stimulus video clips, which provides crucial information about sign onset and offset, as well as about the amount and location of motion occurring in the stimulus clip.

## Methods

### Participants

Ratings for iconicity, AoA, and frequency were obtained from deaf signers in Leipzig, Göttingen, and Hamburg. Guesses about the potential meaning of a sign (from which we then compute transparency scores) and ratings for iconicity were obtained from hearing non-signers in Leipzig. The data from one deaf signer was excluded from the analysis and dataset presented here because they acquired DGS only after puberty (at 17 years of age). One hearing participant was removed from the analysis and dataset because they did not complete all tasks. Hence, the final pools of participants consisted of 32 deaf signers (18 female, 14 male; M age = 40.50 years, SD = 12.39 years) and 30 hearing non-signers (15 female, 14 male, 1 other; M age = 26.03 years, SD = 4.83 years). Deaf and hearing participants were recruited from institutional participant databases, as well as by distributing advertisements at local deaf clubs and organizations, on the internet, and via the authors’ personal contacts.

#### Deaf signers

Most of the deaf participants reported that they were born deaf (*N* = 19) or had become deaf early on in life (i.e., before 3 years of life; *N* = 3). The average self-reported age of sign language acquisition was 3.92 (SD = 3.51), with 13 deaf participants reporting to have acquired DGS from their parents or siblings, and 19 participants indicating to have acquired DGS in a kindergarten and/or school setting. Participants rated their DGS skills as 6.23 on average (SD = 0.84) on a 7-point scale. Most signers indicated that their everyday signing mostly conformed to the local DGS variants used in Hamburg (*N* = 7), Berlin (*N* = 5), North Rhine-Westphalia (*N* = 5), Saxony (*N* = 5), and Lower Saxony (*N* = 3). All deaf participants reported that DGS was their primary as well as preferred mode of communication, with only a few participants (*N* = 9) listing either *Lautsprachbegleitende Gebärden* (LBG, Signed German), spoken and written German, or International Sign as equivalent to DGS in terms of their primary and preferred language of communication. In addition to DGS, deaf participants most frequently reported varying levels of proficiency in ASL (*N* = 22) and International Sign (*N* = 13). Lastly, deaf signers self-reported that on average they had learned to read when aged 5.52 years, with participants rating their current reading skills on average as 5.45 (SD = 1.31) and their current writing skills as 5.10 (SD = 1.16) on 7-point scales.

#### Hearing non-signers

All hearing participants reported that they were native speakers of German and had no knowledge of DGS. No hearing participant reported any knowledge of any other sign language, except for one participant who indicated familiarity with a few signs (i.e., less than 10) from ASL. With regard to spoken and written languages, almost all hearing participants (*N* = 29) reported some knowledge of English as a second language. Other indicated second languages were French (*N* = 9), Spanish (*N* = 5), and Russian (*N* = 3).

### Materials

We initially compiled a large set of about 500 lexical DGS signs. This set of signs was primarily drawn from norming studies for other sign languages (Caselli et al., 2017; Vinson et al., 2008) and amended with further signs considered common by the authors. Our primary goal in this study was to create norms for distinct lexical signs which behave analogous to words in spoken and written language in the sense that they establish a relation between signifier and signified that may be arbitrarily or iconically motivated. We excluded signs based on the manual alphabet (e.g., WC); compounds that may be loan translations from spoken German (e.g., LUNCH, *noon + meal*); number signs; proper names of cities, German federal states, and countries; as well as most classifiers (though consider that our set includes some verbs in their citation form which may incorporate object classifiers by change of handshape during natural language use, for example, EAT; Zwitserlood, 2012). Given that DGS belongs to the group of sign languages that have a special class of so-called agreement verbs which change their path movement and/or hand orientation in order to overtly express agreement between the verb and one or two of its arguments in the signing space in front of the signer (Pfau et al., 2018), we made sure to also include a number of these verbs in their citation form (i.e., without any agreement morphology) in the data set. In sum, this process enabled us to ensure that (i) all signs in our set were lexicalized signs and (ii) signs varied in familiarity and AoA across the set as established by previous norming studies.

Drawing on these resources, the deaf and hearing authors as well as two informants identified the subset of 313 signs (310 test items and three practice items) that was used in this study on the basis of the following three main criteria:

1. The first criterion was the avoidance of polysemy and homonymy in order to ensure that the collected ratings by deaf participants actually reflected values for the target lexical items instead of a sign with a related meaning or formationally similar or identical sign with a different meaning. The deaf and hearing authors and our informants independently assessed the potential for polysemy and homonymy of every sign in the initial stimulus set.
2. Because DGS, like many sign languages, does not overtly mark lexical category by derivational morphology in the case of nouns and simple verbs (Meir, 2012; though consider the personal agreement marker [PAM]; e.g., Rathmann, 2003; Steinbach, 2011), a considerable number of signs may be assigned either a nominal or verbal interpretation solely depending on syntactic context (i.e., similar to English *a book* and *to book*, the DGS sign WORK may be interpreted as *the work* or *to work*). Consequently, we also considered a sign’s homonymic potential with regard to lexical categories. The goal was to include a similar number of signs with and without this special kind of ambiguity in the set.
3. The third criterion was the exclusion of signs belonging to semantic fields which are known to exhibit significant regional variation in DGS such as, for example, signs for weekdays and months (Langer, 2018). More generally, we aimed at minimizing the potential degree of dialectal variation in our set of signs to ensure that derived norms can readily be employed for studies of DGS throughout Germany without introducing a potential confound in the form of little-known or unknown signs due to regional variation.

The final set of 313 signs was recorded on video with one of the authors of this paper who is deaf and a native DGS signer in the professional filming facilities of the SignLab at the University of Göttingen. Three of these signs were used for practice trials so that the final set of normed signs consisted of 310 signs. Figure 1a–d shows representative still images from the recorded stimulus video clips for four DGS signs with varying iconicity and transparency. Because mouthing is common in DGS and may even serve to distinguish minimal pairs such as MARMELADE and COLOR (Boyes Braem, & Sutton-Spence, R. (Eds.)., 2001), the use of mouthings alongside signs was not prevented or suppressed during filming. Similarly, the video clips also includelexicalized nonmanual components (Pendzich, 2018, 2020). Accordingly, the signer was instructed to produce mouthings and nonmanuals spontaneously in the same manner as they would produce them in normal signed discourse.

### Procedure

Because data was collected on site in Leipzig, Göttingen, and Hamburg, the experimental procedure for both groups of participants was implemented in the survey system LimeSurvey version 2.73.1 [Computer software] (Limesurvey GmbH, 2018) to ensure that the course of events and the instructions given were consistent across labs and participants. In compliance with the European General Data Protection Regulation, the LimeSurvey installation and the responses collected from participants were hosted on secure servers of the Max Planck Society located in Göttingen, Germany. All participants were invited into one of the labs in order to perform the tasks on a computer in the presence of an experimenter who communicated with them exclusively in their preferred language (i.e., DGS for deaf signers and German for hearing non-signers). We chose to collect data on site instead of online because (i) online rating data seems to require larger sample sizes in order to obtain data of a comparable quality to data collected in the lab (Wurm & Cano, 2011) and (ii) the projected overall duration of the experiment for both groups would likely have led to participants dropping out.

#### Deaf signers

General instructions, consent information, and specific task instructions were given to participants in DGS. In addition, to ensure that all participants receive similar instructions, we used pre-recorded videos in DGS which were shown on screen before the start of the experiment and before the start of every particular task. These videos describing the experiment as well as the different tasks in DGS are available as part of the supplementary material. Furthermore, the German and English correspondence of all task instructions given to participants in DGS via video is also available as part of the supplementary material. Written informed consent was obtained from all participants before the start of the experiment. Participants received monetary compensation regardless of whether they completed all tasks. In addition, the experimenter double-checked that participants had understood task instructions.

Deaf signers performed three consecutive tasks on our complete set of signs in that particular order: (1) In the first task, participants rated the iconicity of the respective sign on a scale from 1–7 (1, *not iconic*; and 7, *very iconic*). (2) In the second task, participants were asked to indicate the age at which they believe to have acquired a particular sign on a scale from 0–2 to 17+. Following Vinson et al. (2008), the complete scale included the following age ranges: 0–2, 3–4, 5–6, 7–8, 9–10, 11–12, 13–14, 15–16, and 17+. (3) In the final task, participants were then asked to rate how often they use and see a particular sign on a scale from 1–7 (1, *hardly ever*; and 7, *very often*). This arrangement of tasks ensured that participants would not use the same scale back-to-back for rating different constructs (i.e., iconicity, AoA, and frequency). In all three tasks, each screen showed the video of the target sign on a white background, as well as the appropriate scale underneath. In addition to the responses on the different scales, participants could select a “?” response in order to indicate that they did not know a particular sign. Items were always presented in an individually randomized order. Participants could perform each task at their own pace and were given the opportunity to take breaks after completing a task. To ensure that participants perform a task correctly, every task was preceded by three practice trials in which participants were asked to rate the signs FUN, BIRTHDAY, and THEATER on the respective scale. These signs were not included in the final analysis.

#### Hearing non-signers

The implementation of the experimental procedure for hearing non-signers relied on the same software and infrastructure as those used for deaf signers. All hearing non-signers were invited to come to the lab in Leipzig in order to perform the tasks on a computer in the presence of an experimenter. Instructions were given on screen in written German. A copy of these task instructions given to participants in German and a translation into English is available as part of the supplementary material. Again, written informed consent was obtained from all participants before the start of the experiment. Participants received monetary compensation regardless of whether they completed all tasks. The experimenter double-checked that the participants had understood task instructions.

Hearing non-signers performed two consecutive tasks in that particular order: (1) In the first task, participants guessed the meaning of a particular sign. They were instructed to type their response (preferably a single word) into the text box underneath the sign. Another optional box made it possible for them to provide a rationale for their guess. In the present paper, we only analyze the data obtained from participants guesses. (2) In the second task, participants were asked to rate the iconicity of the respective sign on a scale from 1–7 given the sign’s most likely meaning (1, *not iconic*; and 7, *very iconic*). In addition to this rating on the scale, participants were optionally asked to indicate why they assigned a high rating to a sign that they believed was clearly iconically motivated. These qualitative data are also not included in the present analysis. Identical to the procedure for deaf signers, each screen showed the video of the target sign on a white background as well as the appropriate scale or input box underneath during both tasks. Items were always presented in an individually randomized order. Participants could perform each task at their own pace and were given the opportunity to take breaks after rating the first 155 signs in the first task as well as after completing the first task. To ensure that participants perform a task correctly, both tasks were preceded by three practice trials in which participants were asked to perform the task for the signs FUN, BIRTHDAY, and THEATER. These signs were not included in the final analysis.

### Data analysis

Ratings for the following lexical variables variables were analyzed: Iconicity, AoA, and frequency for deaf signers; iconicity and transparency for hearing non-signers. The data for every task was extracted from LimeSurvey per participant in CSV format. All further data cleaning, reshaping, and analysis was carried out using R version 3.4.4 [Computer software] (R Core Team, 2019). The complete reproducible R code used for all analyses reported in this paper is available as part of the supplementary materials (directory name “analysis”).

#### Individual lexical variables

For all tasks employing a rating scale—iconicity, frequency and transparency—we computed the mean per sign using the corresponding scale (Caselli et al., 2017; Vinson et al., 2008). For the AoA task performed by deaf signers, we converted the ratings from ranges into numeric values prior to analysis following the procedure described by Vinson et al. (2008). That is, we converted all intermediate values to the midpoint of the range (e.g., “age 3–4” was converted into 3.5), whereas the endpoints of the scale were treated differently (i.e., “age 0–2” was assigned a value of 1.5 and “age 17+” was assigned a value of 17.5). Transparency of the DGS signs in our set to hearing non-signers with no experience of DGS or any other sign language, was computed using a proximity transparency score capturing the percentage of correct guesses by participants. Specifically, a response was considered correct if the typed word (regardless of case or spelling) corresponded to one of the possible translations of the sign into German or one of the given homonyms (if applicable). As a sanity check, intra-class correlations (ICC) were computed for all tasks that employed a rating scale using the “psych” package version 1.8.12 (Revelle, 2018). We selected ICC(2,k) as the model that is most suitable to determine how well our mean ratings per sign in different tasks generalize to the entire population of raters (Shrout & Fleiss, 1979). Where appropriate, comparisons to other published sign language data were performed using the “cocor” package version 1.1-3 [Computer software] for comparing correlations (Diedenhofen & Musch, 2015).

#### Correlations between lexical variables

We used the “stats” package included in R to compute Pearson’s product-moment correlations. The magnitude of relationship was calculated for the following pairs of variables: (1) iconicity (hearing non-signers) – transparency (hearing non-signers); (2) iconicity (deaf signers) – transparency (hearing non-signers); (3) iconicity (deaf signers) – iconicity (hearing non-signers); (4) iconicity (deaf signers) – AoA; (5) iconicity (hearing non-signers) – AoA.

#### Motion-tracking information

Movement parameters (25 points on the body, including head, arms, torso, and legs) for the different video clips were derived by fitting the BODY25 model using OpenPose version 1.2 [Computer software] (Wei, Ramakrishna, Kanade, & Sheikh, 2016). Further processing of these data and plotting was performed in R using “OpenPoseR” package version 0.2 [Computer software] (Trettenbrein & Zaccarella, under review; available from https://github.com/trettenbrein/OpenPoseR). Sign onset and offset were automatically coded as follows: for sign onset, we selected the first frame of the first time window in the clip in which five consecutive frames exhibit a Euclidean norm of the sums of velocity vectors of all points in the body pose model above a motion threshold of 150 units. Similarly, sign offset was determined by taking the last frame of the last five consecutive frame above said threshold. This automated procedure aligns with the so-called longer view of the sign which includes transitional movements (Jantunen, 2015).

## Results and discussion

In the following, we first describe the distribution of the different ratings for iconicity (deaf signers), AoA (deaf signers), frequency (deaf signers), transparency (hearing non-signers), and iconicity (hearing non-signers). These discussions include an assessment of the generalizability of the average ratings of a variable per sign to the entire population of raters as captured by ICC. Second, we examine the relationships of some of the different variables in our dataset to, for example, determine the relationship between iconicity and transparency by means of correlation analysis. Throughout our discussion we include comparisons to published data for other sign languages insofar as available and appropriate. Lastly, we provide a brief discussion of the machine-readable information provided with the dataset as well as an illustration of the information derived from automated motion tracking.

### Iconicity (deaf signers)

**Fig. 2:**
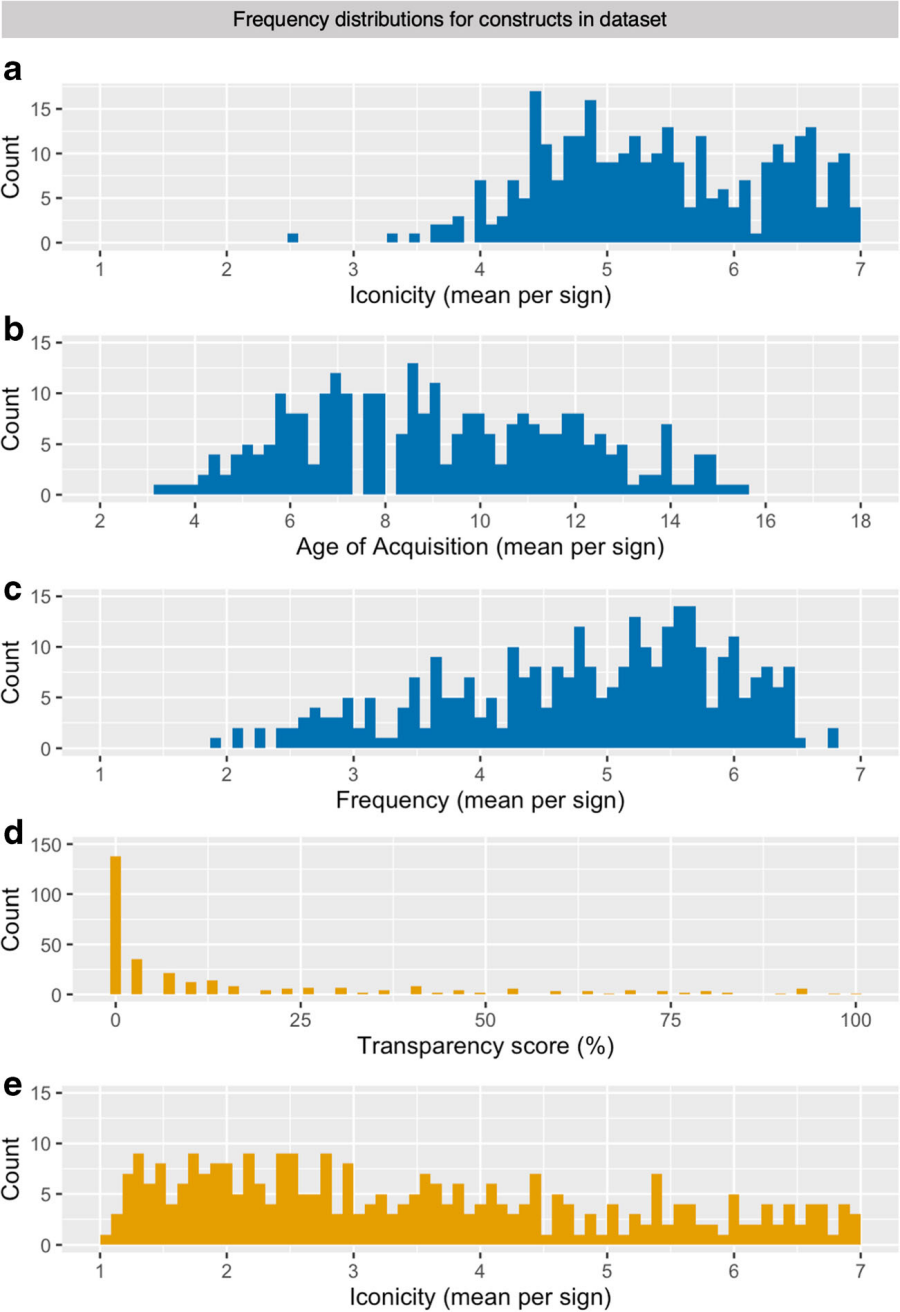
Frequency histograms showing the distribution of ratings for the 310 DGS signs normed in the present study. Results from the group of deaf signers are color-coded in blue, those from hearing non-signers are presented in orange. **a** Distribution of iconicity ratings obtained from deaf signers. **b** Distribution of age of acquisition ratings obtained from deaf signers. **c** Distribution of frequency ratings by deaf signers. **d** Distribution of transparency scores for each sign computed from guesses of a sign’s meaning by hearing non-signers. **e** Distribution of iconicity ratings obtained from hearing non-signers

Iconicity ratings by deaf signers were skewed to the higher end of the scale (Fig. 2a). This result differs from the distribution of iconicity ratings by deaf signers reported by Vinson et al. (2008) where ratings were more evenly distributed and only slightly skewed to the lower end of the scale. Notice, however, that the overlap of our final dataset with the set used for the BSL norming was less than 50% (as determined by automatically comparing BSL glosses to possible English translations of DGS signs). In our dataset, BOOK (M iconicity = 7.00; see Fig. 1a), SCISSORS (M iconicity = 6.97), and SLEEP (M iconicity = 6.97; see Fig. 1b) are the most iconic signs. Indeed, these signs can be considered transparently iconic insofar as it can reasonably be assumed that their meanings can easily be guessed, even by non-signers (Lieberth & Gamble, 1991). Interestingly, however, only SLEEP was 100% transparent to our group of hearing non-signing participants, whereas SCISSORS (transparency score = 46.67/100) and BOOK (transparency score = 53.33/100) were only guessed correctly by half of the participants. Given that we collected such guesses of a sign’s potential meaning for all signs in our set, the relationship between iconicity and transparency will be examined further below. On the other extreme of the distribution, BOY (M iconicity = 2.48; see Fig. 1c), LIE (M iconicity = 3.33; see Fig. 1d, and UNCLE (M iconicity = 3.46) received the lowest iconicity ratings according to deaf signers. Fittingly, the meaning of all three signs was never guessed correctly by any of the hearing non-signing participants. Lastly, the average measure ICC(2,k) = .86 with a 95% confidence interval from .82 to .89 (F(309,9579) = 13.8, *p* < .001). That is, the ICC for average ratings of iconicity per sign generalized to the entire population of raters can be considered good or even excellent by conventional standards (Cicchetti, 1994).

### AoA (deaf signers)

Mean ratings for AoA are distributed across the scale yet slightly skewed to the lower end, which is earlier acquisition (Fig. 2b). The signs with the lowest AoA ratings were EAT (M AoA = 3.34), RABBIT (M AoA = 3.97), SLEEP (M AoA = 3.59), and TOOTHBRUSH (M AoA = 3.75). The signs LECTURER (M AoA = 15.47) and SMARTPHONE (M AoA = 15.31) received the highest ratings. In this context, it is important to note that sign language acquisition may be delayed in cases where deaf children were not offered sign language input early in infancy and have only been exposed DGS in a day nursery, kindergarten, and/or school setting (Meier, 2016; Quer & Steinbach, 2019). The average self-reported mean age of sign language acquisition (3.92 years) of our participants is therefore reflected in the minimum and maximum means of signs in the sample, as well as the shape of the distribution. Similar to the distribution of AoA ratings by Vinson et al. (2008), the distribution of our ratings reflects this on average later onset of sign language acquisition in contrast to similar data for spoken languages (e.g., Schröder et al., 2012). Notice that these ratings should hence not be considered a valid estimate of the objective AoA, that is the actual age at which any sign may be expected to be acquired. Instead, these ratings should primarily be considered as capturing the relative AoA of a sign in relation to all the other signs in the dataset. This is especially true with regard to the AoA ratings for signs such as SMARTPHONE already mentioned above, but also for INTERNET (M AoA = 14.44) and DVD (M AoA = 13.92), as the ratings for these signs are evidently influenced by the average age of participants (i.e., for older participants the internet or DVDs did not yet exist when they grew up). Regarding internal consistency of the scale, the average measure ICC(2,k) = .94 with a 95% confidence interval from .92 to .96 (F(309,9579) = 22, *p* < .001). In other words, the ICC for average ratings of AoA for every sign generalized to the entire population of raters can be considered excellent according to established classifications.

### Frequency (deaf signers)

For the frequency task, the mean ratings by participants were skewed to the higher end of the scale (Fig. 2c), suggesting that the vast majority of signs in our set are well known and frequently used amongst DGS users. This assessment is confirmed by the fact that the “?” option indicating that the participant did not know a sign was only used extremely sparsely by participants (about 1% of the responses for this as well as all tasks in total). This indicates that we successfully managed to ensure broad familiarity and limited dialectal variation, as was the goal of our selection for signs to be included in our set outlined above. The signs with the lowest frequency ratings were CAPTAIN (M frequency = 2.07), NUN (M frequency = 1.93), and PIPE (M frequency = 2.09). The signs with the highest frequency ratings were EAT (M frequency = 6.75) and GOOD (M frequency = 6.81). Again, the average measure ICC(2,k) = .93 with a 95% confidence interval from .92 to .95 (F(309,9579) = 37, *p* < .001) which can be considered excellent by conventional standards.

### Transparency (hearing non-signers)

A first look at the distribution of transparency scores already reveals an extreme skew to the left (Fig. 2d). In other words, almost half of the signs in our set (44.52%) were never guessed correctly by any of the participants. Given that our stimulus clips included nonmanuals and natural mouthings, it is reasonable to disregard signs with a transparency score below 7/100 (i.e., less than at least three correct responses by hearing non-signing participants), which could potentially reflect the participants ability to lipread. Accordingly, only 34.83% of the signs in our set elicited more than two correct responses. Similarly, only 11.61% of all signs elicited correct responses by the group of hearing non-signers more than 50% of the time. The signs SLEEP (transparency score = 100/100) and PHOTO (transparency score = 96.67/100) were the two most transparent signs. No sign other than SLEEP was guessed correctly by all participants. These most transparent signs also received very high iconicity ratings (M iconicity > 6.8) by the group of deaf signers. In sum, these transparency scores provide a means for researchers to quantify the likelihood of a DGS sign being transparent to participants without any knowledge of DGS or any other sign language.

### Iconicity (hearing non-signers)

In addition to iconicity ratings by deaf signers, we also collected iconicity ratings from the group of hearing non-signers. By collecting iconicity ratings from both groups of participants we wanted to characterize group differences with regard to the awareness of iconicity that may result from knowledge of DGS or sign language knowledge in general. For this task performed by the hearing non-signing participants, mean ratings are distributed across the scale yet clearly skewed to the lower end (Fig. 2e). Recall that this is in direct opposition to the distribution of iconicity ratings collected from deaf signing participants (Fig. 2a). Consequently, it seems that knowledge of sign language has an impact on participants’ awareness about iconicity. Interestingly, the distribution of mean iconicity ratings observed here resembles the spread and skew of the distribution of a larger set of iconicity ratings for ASL collected online from a group of hearing non-signers by Caselli et al. (2017). These observations raise the question of the relationship of ratings collected with our deaf and hearing groups which we will discuss below. Again, similar to the different ratings of the same construct by deaf signers, the average measure ICC(2,k) = .97 with a 95% confidence interval from .97 to .98 (F(309,8961) = 45, *p* < .001). That is, according to conventional standards, the ICC for average ratings of iconicity per sign rated by hearing non-signers generalized to the entire population of raters can be considered excellent.

### Relationship between iconicity and transparency

**Fig. 3:**
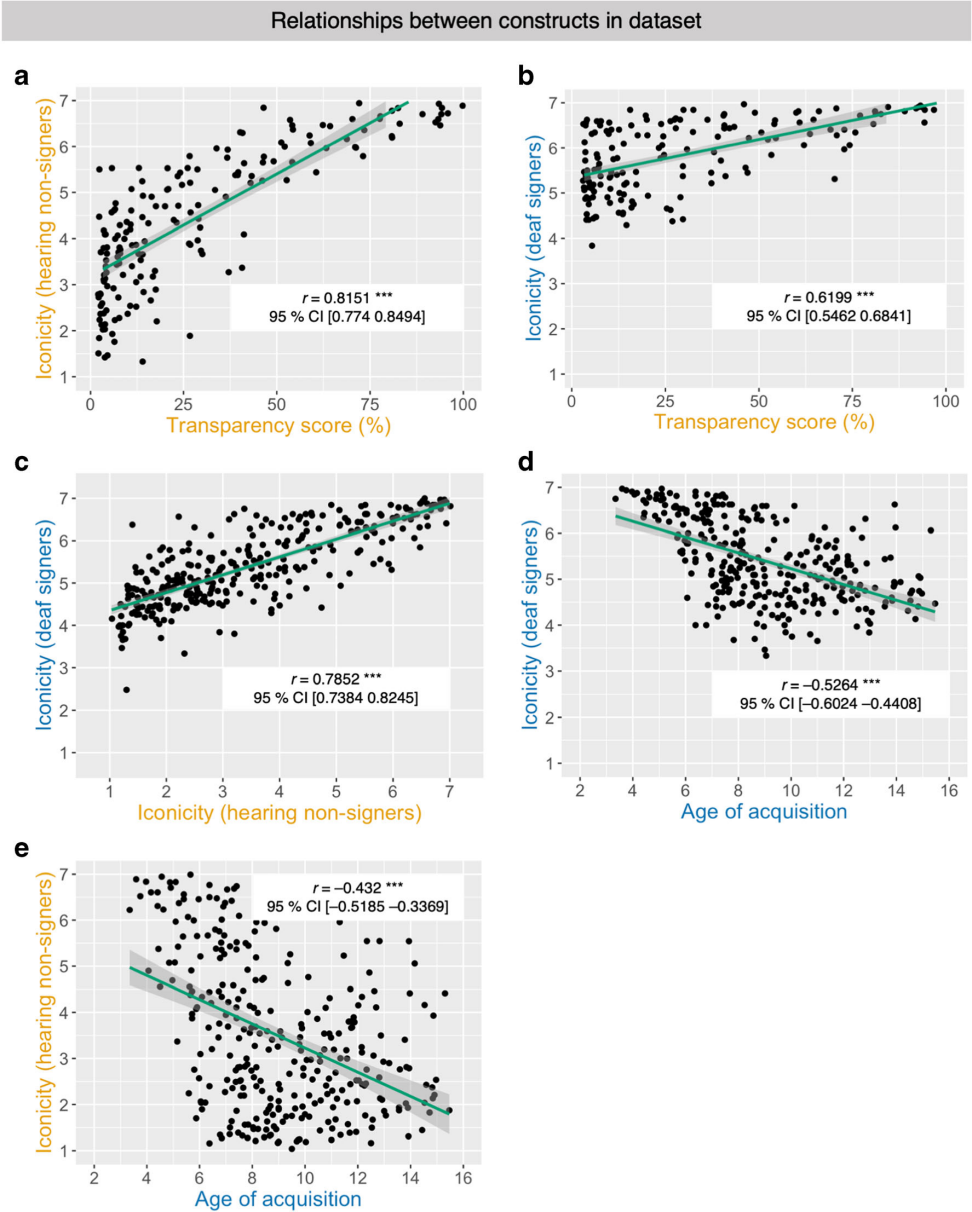
Scatterplots and regression lines (with 95% confidence intervals) depicting the correlations between different variables in the dataset. **a** Transparency scores by hearing non-signers and iconicity ratings by hearing non-signers, **b** transparency scores by hearing non-signers and iconicity ratings by deaf signers, **c** iconicity ratings by both groups of participants, **d** iconicity ratings by deaf signers and ratings for AoA by deaf signers, and **e** AoA ratings by deaf signers and iconicity ratings by hearing non-signers

This issue of the relationship between iconcity and transparency was already discussed by Klima et al. (1979) and, amongst others, investigated further by Emmorey (2014). Throughout this discussion in the literature, one of the fundamental ideas has been that signs with higher iconicity ratings should in general be more transparent to non-signers than signs with lower iconicity ratings (Lieberth & Gamble, 1991). When presupposing that iconicity is the driving force of a sign’s transparency, we should expect that transparency scores and iconicity scores are correlated. In other words, signs that were more transparent to non-signers should also have higher iconicity scores and vice versa, provided that participants’ correct guesses of a sign’s meaning were enabled by the sign’s iconic motivation. Transparency scores and mean iconicity ratings by hearing non-signers were indeed highly correlated (Fig. 3a): *r* = 0.82, 95% confidence interval from 0.77 to 0.85 (*p* < .001, coefficient of determination: *r*^2^ = 0.66). Hence, there is a strong relationship between iconicity and transparency within the group. That is, the non-signing participants assigned higher iconicity ratings to signs in the second experimental task if those signs previously had been more transparent to them during the first task.

Because we also collected iconicity ratings from our group of participants who are deaf signers, we could also explore the effect of sign language knowledge on the relationship between iconicity and transparency. Iconicity ratings by deaf signers and transparency scores by hearing non-signers are also significantly correlated (Fig. 3b): *r* = 0.62, 95% confidence interval from 0.55 to 0.68 (*p* < .001, coefficient of determination: *r*^2^ = 0.38).This suggests that there is a moderately linear relationship between a sign’s iconicity as judged by deaf signers and the transparency of a sign to non-signers. However, sign language knowledge is required in order for participants to be able to correctly identify and reliably establish the conventionalized structured mapping between the salient property of a referent (e.g., the DGS sign BOOK depicting the opening of the pages of a book; Fig. 1a) and the actual lexical meaning *book*. This is reflected in the fact that, for example, the sign BOOK only received a transparency score of 53.33/100, despite receiving very high iconicity ratings by deaf signers (M iconicity = 7) as well as hearing non-signers (M iconicity = 6.57). Comparing the values of *r*^2^ for both groups shows that only 38% of the variation is related across both groups, but 66% of variation is related within the group of hearing non-signers. This shows that knowledge of sign language had a significant impact on our participants’ awareness of iconicity. In lack of sign language knowledge, hearing non-signers were less sensitive to a sign’s iconic motivation and instead drew upon supposedly iconic cues such as handshape or location for their guesses of a sign’s potential meaning (e.g., guessing *beard* or *mustache* based on the handshape and location of the sign CAT).

### Relationship between iconicity ratings across groups

Next, we further explored the relationship between iconicity ratings by our groups of deaf signers and hearing non-signers. As already briefly mentioned above, one possibility is that deaf signers tend to consider signs to be “more iconic” than hearing non-signers because of their sign language knowledge. This actually seems to be the case when looking at the distribution of the ratings in the two groups (see Fig. 2a and e). Yet, despite these differences in awareness with regard to iconicity, we would still assume iconicity ratings across groups to be strongly correlated, because iconicity ultimately is not an issue of subjective judgment but a strategy for motivating lexical signs that iconically signify the same or at least a very similar pre-linguistic concept. Indeed, iconicity ratings by deaf signers and iconicity ratings by hearing non-signers are significantly correlated (see Fig. 3c): *r* = 0.78, with a 95% confidence interval from 0.74 to 0.82 (*p* < .001, coefficient of determination *r*^2^ = 0.62). This strongly suggests that deaf signers and hearing non-signers actually judge a sign’s iconicity using a similar strategy, despite the fact that their use of the scale results in differently skewed distributions. Accordingly, sign language knowledge seems to increase the signers’ sensitivity towards iconicity in a manner that is not available to non-signers. A possible reason for this increased sensitivity towards iconicity is that signers may have knowledge of a sign’s etymology. That is, they may be more prone to still consider signs as iconic which have lost some or all of their initially iconic properties in the process of language change (e.g., the now “opaque” ASL sign HOME; Klima et al., 1979).

The observation that signers and non-signers judgments of iconicity are highly correlated seems to hold up cross-linguistically: In a study on ASL with a similar setup, Sehyr, Fisher, and Emmorey (2017) report analogous results for a comparison of deaf signers and hearing non-signers. A statistical comparison of the observed correlations for DGS and ASL reveals that Fisher’s z = – 1.25, with a 95% confidence interval from – 0.09 to 0.02 (*p* > 0.05). This points to a similar relationship of subjective iconicity in signers and non-signers for both sign languages. However, different from the study on ASL, both groups in the present study exhibited different biases in using the rating scale: Deaf signers on average tended to use values on the right end of the scale, thereby indicating a bias for considering signs very iconic on average. Hearing non-signers do not show this bias due to lack of sign language knowledge. As already mentioned above, the distribution of iconicity ratings by hearing non-signers fits the wide spread and left-skewed pattern reported for hearing non-signers by Caselli et al. (2017), yet the right-skewed distribution for our group of deaf signers differs from the pattern for deaf signers reported by Vinson et al. (2008) as well as the group comparison reported by Sehyr et al. (2017).

### Relationship between iconicity and AoA

Sign language acquisition is another area in which iconicity has been hypothesized to potentially play a role. Given that iconicity can aid sign language acquisition for adults in the context of second-language learning (Ortega, 2017), one might speculate as to whether signs which are on average acquired earlier during first language acquisition tend to be more iconic. Meier (2016) cautions that no such relationship should be expected, given that (i) many signs which are acquired early in life are not iconic (e.g., the DGS sign BOY; see Fig. 1c) and (ii) recognizing the iconic motivation of a sign like MILK (roughly imitating the milking of a cow with both hands) requires knowledge about the dairy industry, which is available to adult learners in a second language context but not to infants during language acquisition. Accordingly, we analyzed the relationship between iconicity ratings by deaf signers, hearing non-signers, and the ratings for AoA in our dataset. We found that iconicity ratings by deaf signers and AoA ratings are significantly negatively correlated (see Fig. 3d): *r* = – 0.53, 95% confidence interval from – 0.60 to – 0.44 (*p* < .001, coefficient of determination: *r*^2^ = 0.28). This is a first indication that there is a moderate relationship between iconicity and AoA ratings in our dataset. Next, we examined iconicity ratings by hearing non-signers and AoA ratings and found that they are also significantly negatively correlated (see Fig. 3e): *r* = – 0.43, 95% confidence interval from – 0.52 to – 0.34 (*p* < .001, coefficient of determination: *r*^2^ = 0.19). In comparison to the relationship of iconicity and AoA found for deaf signers, the relationship between iconicity ratings by non-signing participants and AoA ratings by deaf signers is weaker. For ratings by non-signers and AoA, only 19% of variation is related, whereas 28% variation is related within the group of deaf signers. This indicates that the group difference in the subjective rating of iconicity we observed does not lead to major differences in relationship for iconicity and AoA for both groups. Because our dataset was not compiled to be representative for a language acquisition context we cannot draw any general conclusions about the relationship of iconicity and AoA in sign language acquisition. However, within our dataset we observe that the relationship between iconicity and AoA is at best weak.

### Machine-readable annotations and motion-tracking information

To make this stimulus set maximally useful for different researchers, the present dataset also includes a variety of information about every sign’s lexico-semantic and phonological properties in a machine-readable format. All signs are annotated as to whether they are articulated using only the signer’s dominant hand (i.e., they are one-handed) or if they are articulated using both of the signer’s hands (i.e., they are two-handed). The primary place of articulation (i.e., close to the head, on the non-dominant hand, on the signer’s body or in sign space in front of the signer) and information about a sign’s most likely lexical class (noun, verb, adjective, or adverb) is also included. Of the 310 signs in the dataset, 67.42% were assigned to only one major lexical class (nouns: 46.13%, verbs: 12.90%, adjectives: 7.42%, adverbs: 0.97%). In cases where syntactic context determines lexical class (32.58% of signs in dataset), signs are coded as belonging to more than one category (e.g., the sign SLEEP shown in Figure 1b could mean either *to sleep* or *the sleep*; or the sign ANSWER which could mean either *to answer* or *the answer*). Because DGS has a special class of so-called agreement verbs which change their path movement and/or hand orientation in sign space to mark object and subject in a sentence, verb class (i.e., agreement verb or plain verb) is also coded in the dataset. For signs with a possible nominal interpretation, we also provide information as to whether the noun would be considered animate or inanimate. Lastly, we also include a list of potential homonyms for every sign (if any), information about common dialectal variation (even though we tried to minimize the number of signs of which we and our informants were aware that they are likely to vary regionally), as well as likely German and English correspondences; even though it should be emphasized that such translations are at best approximations and must be used with due caution.

**Fig. 4:**
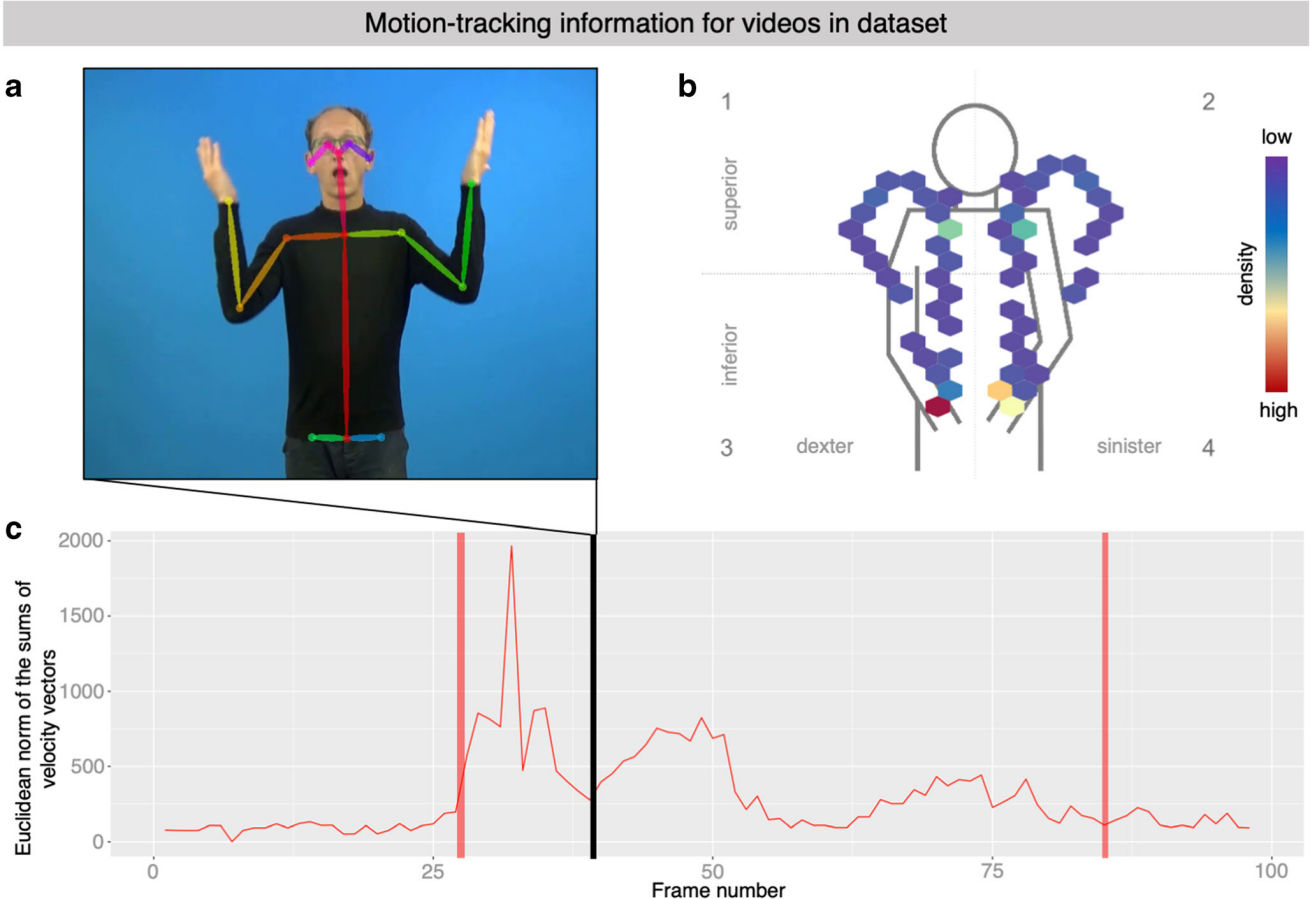
Illustrations of information contained in the motion-tracking data that is part of the stimulus set. **a** Representative frame from the example video clip EVENING with the fit body pose model from which motion tracking data is derived. **b** Location information for the two main articulators used to produce the sign EVENING (i.e., left and right hand) throughout the video clip. *Colors* indicate density from low (*violet*) to high (*red*). The symmetry of the sign EVENING as well as the hold in front of the chest at the end of the sign is clearly visible. **c** The Euclidian norm of the sums of velocity vectors is used to quantify the amount of movement by the signer in the stimulus clip for EVENING. The *black line* indicates the timing of the representative frame shown in **a**. *Red lines* indicate timepoints of sign onset and offset for this video clip as automatically computed from motion tracking data, in accordance with the so-called longer view of the sign (Jantunen, 2015)

In addition, the dataset also includes information from automated motion-tracking derived by fitting a body-pose model for every video clip (example frame with fit model in Fig. 4a). Location information derived from this model can be used to track the two main articulators for the sign EVENING (i.e., left and right hand), for example, which is shown throughout the video clip in Fig. 4b, whereas colors indicate density from low (violet) to high (red). The symmetry of the two articulators as well as the final hold in front of the chest at the end of the sign is clearly visible. The availability of this information also makes it possible to quantify the amount of movement occurring at a certain point in time in a video clip and was used to automatically determine sign onset and offset in a purely data-driven manner (Fig. 4c, color-coded in light red), in accordance with the so-called longer view of the sign (Jantunen, 2015). We suggest that this detailed information about the provided stimulus video clips can, for example, be useful to researchers when designing studies that require close attention not only to a sign’s psycholinguistic properties, but also to the signer’s different movement parameters recorded in the stimulus video clips such as, for example, the visible movement of articulators and even more so the movement and position of the signer’s body as a whole. The raw data resulting from these model fits is also provided alongside this normed dataset and allows for the implementation of individual analyses and means for stimulus control.

## Conclusions

With ratings for iconicity, AoA, frequency, and transparency, as well as the machine-readable annotations and information from automated motion-tracking, our normed stimulus set provides an opportunity for sign language researchers working on DGS to construct experiments in which a number of lexical variables can be controlled for the very first time. In the future, the availability of large-scale corpus data for DGS will hopefully make it possible to supplement the subjective rating data for lexical frequency presented here with quantitative measures. At the same time, subjective ratings of AoA, iconicity, and transparency will remain indispensable. We acknowledge that the dataset described here is limited in terms of the number of signs included, sample size, and geographical distribution of participants. Yet, we hope that making these norms publicly available through the Open Science Framework may prove to be useful to other researchers carrying out studies using DGS as well as other sign languages. Also, we encourage likeminded researchers to build upon and expand this dataset by complementing it with additional measures or more signs.
